# Formative research and design for a mobile health platform for oral cancer screening and detection (OC-DETECT)—a mixed methods study

**DOI:** 10.3389/fdgth.2025.1738874

**Published:** 2026-02-02

**Authors:** Krystyna R. Isaacs, Praveen N. Birur, Mariam Siddiqui, Kajal Patel, Aarenee I. Greene, Lopamudra Ray Saraswati, Jigyasa Singh, Yukiko Washio, Yi Cui, H. Katie Chang, Tony X. Ma

**Affiliations:** 1 Benten Technologies, Manassas, VA, United States; 2 Department in Oral Medicine and Radiology, KLE Society's Institute of Dental Sciences, Bangalore, India; 3 RTI International, New Delhi, India; 4 RTI USA, Durham, NC, United States

**Keywords:** digital image collection, education, mobile-phone-based assessments, oral cancer, provisional diagnosis, user-centered design

## Abstract

**Introduction:**

Oral cancer (OC) in India accounts for one-third of the global burden of OC cases and mortality and is the third most frequent cancer in India. This study details formative research conducted to inform the design of a prototype mobile health platform that would consist of a participant-side mHealth screening app and a clinician side (a desktop-facing interface). The initial design included a risk assessment for general health as well as alcohol and tobacco habits, followed by a tool to upload digital images of the oral cavity taken with a mobile phone. The physician could then review the images remotely and make a provisional diagnosis.

**Methods:**

E-Surveys were distributed to healthcare providers associated with the Indian Cancer Society in New Delhi (*n* = 11) and young people (*n* = 56) attending colleges in New Delhi. Questions were asked about oral health awareness, oral cancer awareness and possible barriers to seeking medical care when oral lesions were detected. Initial focus groups with young people (*n* = 17 individuals) and in-depth interviews with providers (*n* = 6) explored the resistance to visiting a clinic, issues related to trusted communications and educational materials, and willingness to use a mobile health application to collect personal health information and digital images. Second and third round of interviews and focus groups focused on reviews of low- and high-resolution wireframes of the initial designs before completing a final prototype design.

**Results:**

By utilizing a user-centered design approach, we concluded that young people and providers welcomed the opportunity use mobile phones to detect potential oral lesions in smokeless tobacco users and to seek care for family members, but had some concerns about issues related to privacy and personal health information. Lack of awareness of oral health issues was identified as a major barrier to seeking care, and a lack of access to reliable and trustworthy educational materials contributed to this problem.

**Conclusions:**

As a result of this formative research, a final prototype is presented to produce a mobile health platform for the detection of oral cancer (OC-DETECT) which will then be tested at dental camps in New Delhi administered by the Indian Cancer Society.

## Introduction

Oral cancer (OC) in India accounts for more than one-third of the global burden of OC cases and mortality (1) and is the second most frequent cancer in India (1). OC forms in tissues of the oral cavity or the oropharynx (2) and is strongly linked to tobacco use (smoking, vaping, or chewing) and is exacerbated by heavy alcohol use (3). Consumption of smokeless tobacco (SLT) is more prevalent than various smoking tobaccos in India (4). Approximately 23% of all males in India using SLT by chewing, applying it to the teeth and gums, or by sniffing (4). Prevalence rates for lip and oral cavity cancer are predicted to increase through 2040 (5).

There are multiple barriers to the detection of oral cancers in India. Low-wage earners are the most frequent users of SLT. These individuals are restricted from using the public health system due to cost and typically experience significant delays in getting an appointment and long wait times at the clinic (approximately 2–3 h) (6). As such, a trip to the health center can mean significantly reduced earnings on that day. In addition to the potential heavy caseload for each dentist in Delhi (over 2,500 patients/dentist) (7), many individuals only see the dentist if they experience pain or discomfort, not for regular dental cleanings (8) and the screening rates for OC in Indian males is approximately 1.0% and 0.053% in Indian females (9). Another significant barrier is the lack of trained professionals to diagnose early stage OC and potentially malignant disorders. One outcome of these challenges is that more than half of oral cancers in India are detected when they are in the most advanced stages (10)—when effective treatment is complicated.

To address the growing burden of non-communicable diseases, the Indian government launched a national program focused on strengthening infrastructure, human resources, health promotion, early diagnosis, and treatment (11). However, oral cancer screenings events are still low with only sporadic opportunistic field-based screenings being carried out (12).

A mobile platform can facilitate the task-shifting of screening, provide on-demand training, improve communication between providers and patients, disseminate evidence-based educational materials, and support digital-enhanced referrals (13). mHealth screenings for alcohol and tobacco can be administered periodically by laypersons equipped with a mobile device (13, 14). For those identified as moderate to high risk for any illness, research also shows that referrals and screenings delivered through mobile devices have the potential to improve early detection of illnesses (15–18) and self-management of chronic diseases (19, 20). A recent study demonstrated that laypersons, without prior medical training, can be trained to effectively detect potentially malignant oral disorders (PMOD) and specifically, oral squamous cell carcinomas (OSCC) of the buccal cheek (21, 22). Task-shifting the initial oral cavity inspection to field health workers equipped with mobile phones in India has also proven effective (18). Mobile health applications have the potential to be useful in under-served communities in both rural and urban settings as 5G connectivity is spreading rapidly in India (23).

Education to counteract stigma and promote self-examinations is possible (24). Lack of knowledge related to risk factors, prevention, symptoms, and prognosis can impede early detection amongst at-risk populations (10, 25–28). Prior general audience education campaigns against SLT have not been successful in reducing rates of uptake of tobacco use (29) and recent studies have shown only a modest cessation to smokeless tobacco products (4) after counseling and/or medication (30). However, educational materials have been successfully tested to increase OC detection of lesions by mouth self-examination (MSE) (31). Evidence supports that visual examination for at-risk individuals results in early OC diagnoses and leads to improved survival rates (32, 33). Mathew et al. (34) evaluated the feasibility of MSE using a pictorial brochure to describe OC risk factors, to recognize possible premalignant and malignant OC lesions, and provide a pictorial guide to MSE. The brochures were distributed to tobacco users aged 30 years or older to read and report suspected abnormalities to a local clinic. About 267 users reported to a clinic where 3% had oral cancer and 34% had precancerous lesions. Similarly, Elango et al. (35) evaluated the feasibility of MSE to improve OC awareness for health education and cancer screening. Other studies supported using MSE combined with educational materials to detect OC lesions (36, 37).

It is crucial to expand OC screenings and educational materials to implement a timely, appropriate and efficient treatment that will reduce morbidity and mortality from oral cancer (7, 32, 38, 39). Community health workers (CHWs) in India typically engage in support to maternal and child health services (40), but their role is now being extended to include non-communicable disease services where they are facing unrealistically heavy workloads (41, 42). In addition, the average education level for CHWs in India is 8 years of schooling (43). An alternative to employing CHWs for screening is to task-shift or recruit tech-savvy young family members who have been trained to use a mHealth app to improve access to family medical histories, conduct screenings, and help their elders to confirm appointment setup and adherence after a referral (13, 44).

We propose to develop an innovative platform called Oral Cancer Detect (OC-DETECT) that will leverage secure, cloud-based technology for individuals that supports on-demand education, for early detection, and linkage to care (LTC) through integrated technology with e-screening, micro-learning educational content, and e-referrals. The app can be designed to work offline and uploads of images and two-way chat can be conducted when wifi is available. We hypothesize that it will be feasible for OC-DETECT to provide mHealth services outside a clinical setting for individuals at risk for OC. The short-term public health impact of OC-DETECT will be to increase screenings to detect OC and provide education to reduce carcinogenic exposures via SLT. The mHealth app will provide enhanced outreach between healthcare professionals, at-risk individuals, and their family members, and support early detection and education. In the long term, the number of individuals identified, referred, and linked to care by OC-DETECT could reduce the mortality and morbidity of OC.

## Materials & methods

### Methods

**Figure 1:**
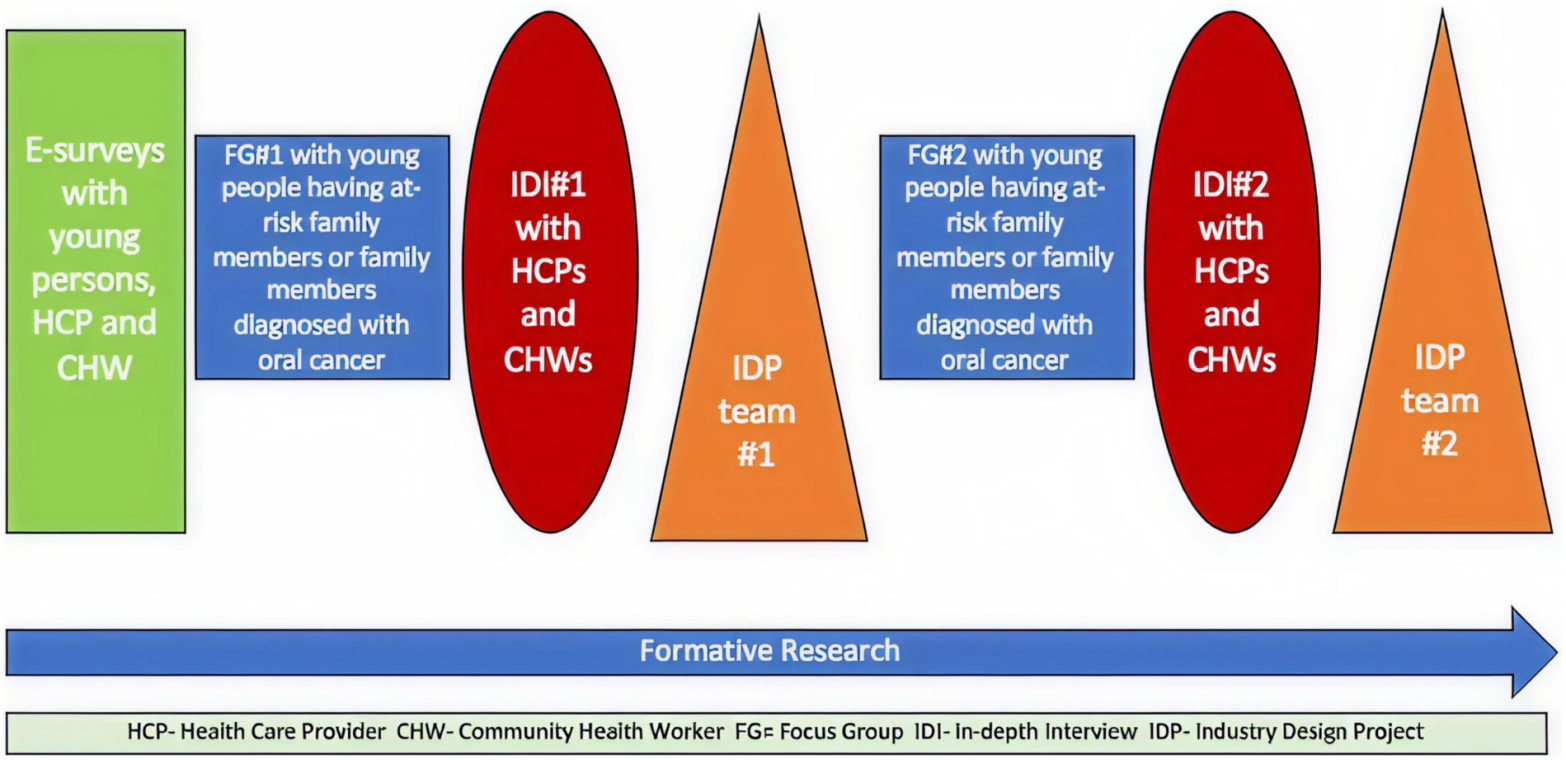
Schematic overview of the formative research design process.

Formative research conducted during the development of the OC-DETECT platform included a combination of e-surveys, focus group discussions (FGDs), and in-depth-interviews (IDIs) to learn more about the key stakeholders' perspective on knowledge about oral cancer, factors affecting screening, and barriers to oral cancer care and a response to early mHealth app designs. This was done prior to commencing the design of the mHealth application (see Figure 1 for a schematic overview of the formative research design). The Indian Cancer Society (ICS) served as the Institutional Review Board (IRB) of record for this research. Health care providers were recruited through the ICS, New Delhi.

### Quantitative methods

#### E-Surveys

Two e-surveys were designed to establish what are the primary issues to be addressed when trying to solve the problem of low screening rates in India, and the lack of prevention programs, one each for healthcare providers (physicians and CHWs and one for students. The e-surveys were produced and distributed through REDCap™ (45) as were the informed consent documents. Individuals were recruited through a health clinic, social media, or through non-governmental organizations that work with communities on cancer prevention and control. Inclusion criterion for the HCP survey was that the providers (physicians and community health workers) needed to work in the ICS dental camps. There were no exclusion criteria for this group. The first online e-survey was designed to learn about healthcare practitioners' and CHWs' opinions on screening and prevention practices. OC-DETECT project staff sent out the e-survey to approximately 100 HCPs and CHWs practicing in the New Delhi region who would be responsible in the future for administering screens and referring individuals to dental health clinics. The second e-survey was sent out to 100 young people between the ages of 18-30 attending universities (Sri Venkateswara College and Jamia Millia Islamia, New Delhi). Inclusion criteria were that the students needed to be enrolled in the university. Individuals who had no regular contact with an elder family member were excluded from the study. The students were invited to answer anonymous e-survey questions about their willingness to perform screenings on other family members, their technological capabilities and their knowledge about oral cancer. All survey respondents were compensated with an e-gift card.

Given the formative aims of the surveys (identifying key barriers, facilitators, and feasibility considerations), we used a pragmatic target sample of approximately 100 respondents per survey. This size supports stable descriptive estimates for prioritization (e.g., worst-case 95% margin of error ∼±10% for proportions) while remaining feasible for rapid needs assessment and intervention planning (46).

All quantitative analyses were done using STATA version 15.1 (College Station, TX). Nothing beyond descriptive statistics, such as frequency distribution and cross-tabulations, was conducted.

### Qualitative methods

#### Focus group with students-session #1

The purpose of FG#1 was to explore e-screenings and electronic referrals with family members of at-risk individuals. Two focus group discussions were conducted with family members. The two groups included a total of 17 students from Sri Venkateswara College in New Delhi in their first year of majoring in either Life Sciences or Biochemistry at the college. Eligibility included: (1) must be between the ages of 18-30; (2) must own a smartphone; (3) must give voluntary consent; (4) be able to read and understand English or Hindi; and (5) must have a family member at-risk for OC or already diagnosed with OC. Informed consent was reviewed and participants signed paper versions of the Informed Consent at the time of the focus group meetings. All participants were assigned an ID # at the time of the session and no personal names were used during the discussion. Subjects were counseled not to comment on the discussions outside of the focus group sessions. Participants were compensated for their time with e-gift cards. Transcripts and audio recordings were examined for items that would influence the design.

#### In-depth interviews with providers-session #1

Community health workers (CHWs) and physicians were interviewed 1:1 to learn more about their opinions about the state of oral cancer care in India. Individuals were recruited by ICS via email solicitations to participate. Eligibility included participants must give voluntary consent, be able to read and understand English or Hindi, and to have regular contact in a primary care setting or dental camp with older individuals at risk for oral cancer. All participants provided written consent for audio recordings and informed consent for participation in the sessions. Participants were compensated. A total of six in-depth interviews with community health workers (CHW, *n* = 3) and physicians (*n* = 3, i.e., dentist, periodontist, and surgeon) were conducted in individual sessions.

#### Analysis

After each focus group session or IDI, a research assistant (RA) used Zoom closed captioning and/or Otter.ai to transcribe the audio recordings. Audio recordings from FGs and IDIs conducted in Hindi were transcribed and translated into English. The transcribed audio file quality was verified by a second RA. We stored the study's qualitative data in Excel tables and created a matrix of questions (columns) by participant/group (rows) and recorded the responses in corresponding cells (47).

Descriptive statistics were used to characterize the participants' sociodemographic characteristics. We considered the discussion data in terms of themes with the goal of noting regularities, patterns, explanations, and propositions contained in the data. We then conducted thematic analysis in which we systematically analyzed the findings from each IDI or FG to identify common themes and similarities and differences. Additionally, we noted substantive quotes as examples of common ideas shared in the responses. Both RAs reviewed the transcripts for themes individually and then compared findings of central themes. Any discrepancies were resolved through discussion between the two RAs.

#### Design round #1

After completion of the first FGDs and IDIs, an Industry Design Project (IDP) team from Springboard (48) was commissioned to create low-fidelity wireframes (pictures of future app screens) using Figma design software. Springboard is a commercial company that trains individuals to be UI/UX designers. These students participated in this research project to complete their portfolios. They were not compensated. The IDP team developed initial design components, including personas, user stories, and user flows. The outcome of this first round included low and high-fidelity wireframes with the multiple features to be incorporated into a prototype platform consisting of three components, one each for the young family member, clinician, and nurse.

#### Focus group students - session #2

The second round of FGDs were designed to solicit feedback on the low-fidelity wireframes produced by the IDP team for the OC-DETECT application. FGD #2 was conducted with 25 students from Sri Venkateswara College, New Delhi and the students were recruited through convenient sampling strategies with the same eligibility criteria as FGD #1. Mockups or low-fidelity wireframes of the OC-DETECT application were used to assess the aesthetics of the design of the app, the relevance of features as it relates to the target population's needs, identify any missing features, assess the readability of content, determine the ease of use of the user interface, and field questions about preferred educational topics and delivery methods. Participants were compensated for their time with e-gift cards.

#### In-depth interviews providers - session #2

The second IDIs were completed by drawing on the same population as the first IDI sessions with CHWs and physicians. Participants were shown wireframes of the first design iteration and asked to comment on the features.

#### Development of evidence-based micro-learning content

The OC-DETECT app included educational materials to improve knowledge about oral cancer and the value of preventive screenings. Short, micro-learning videos (1–5 min) were produced of educational materials capable of being delivered via mobile phone or tablet, with accompanying PDF-formatted handouts available on the app. These team sourced evidence-based peer-reviewed publications (32, 49–55) and scripts were reviewed by subject matter experts (SMEs). Upon completion of the editing, the scripts were sent to a designer for production into short, animated videos set in an Indian community using Vyond^TM^ software.

## Results

### E-survey results

#### Healthcare providers (HCPs)

A total of 11 HCPs participated in the e-survey in May 2023 (Supplementary Table S1). All HCPs thought a lack of oral health awareness is a problem in their community. Most of the HCPs (72.7%) reported that they would annually screen a patient over 40 years of age for oral lesions. When HCPs were asked about perceived reasons that oral health was being under-addressed; most of them reported a lack of oral health awareness (45.5%), followed by a lack of concern for oral health (36.4%). Many of the HCPs (90.9%) thought that young family members could use a mobile app to screen for oral lesions. They thought this to be the case as young people are “more tech-savvy” and “hooked on mobiles and apps” which could facilitate the process of oral screening. Many of the HCPs also thought lack of awareness (45.5%) was a significant barrier in seeking help for oral lesions, followed by lack of facilities (27.3%) and other reasons (27.3%) such as oral health not being a priority, tobacco usage, etc. All HCPs thought there was a need for awareness around oral health, cancer prevention, and treatment. About 70% of the HCPs reported they provide materials on oral health to their patients. In the HCPs' opinion, people get wrong health information mostly from peers, friends, relatives and social media (60%) followed by informal health practitioners (10%) and in lay persons in villages (10%). Finally, in the HCPs' opinion the reasons past tobacco campaigns were unsuccessful included: lack of awareness and information (40%), addiction to tobacco products (20%), lack of political will and control measures along with sale of single cigarettes and cheap chewing tobacco pouches (10%), lucrative advertising by tobacco companies overshadowing campaigns (10%), and campaigns being discouraging in nature (e.g., overly fearful messaging).

#### Young people

We further explored young person's existing oral health knowledge (Supplementary Table S2). Young people recognized several causes of oral lesions, including tobacco use (smoke and smokeless tobacco products) (70%), heavy alcohol use (27%), human papillomavirus (21%), and weakened immune systems (16%). Among the young participants a majority agreed that oral lesions could be prevented (97.6%) and treated (97.6%). Many of the participants also thought the lack of oral health awareness was a problem in their community (92.9%). When asked about where they would go to get credible information about oral health, the following resources were listed: physicians (39%), dentists (39%), government hospitals (27%), mass media (27%), social media (25%), private hospitals or clinics (21%), professional organizations (13%), informal health care providers (11%), Ministry of Health (9%), frontline or community health workers (7%), and nurses (4%).

In addition, because we wanted to utilize young persons for the screenings, we explored the young people's access to mobile phones and network connectivity (Supplementary Table S3) and the use of any health-related apps on a mobile phone (Supplementary Table S4). Many of the participants owned a smartphone (97.9%) and paid used a pay-as-you go service plan (83%). The majority of participants had unlimited data plan (68.1%) and text messaging (59.6%). Many of these participants had access to wi-fi at the university or at home (95.7%). A little over one-third of the participants self-rated the stability, reliability, and coverage of the network connection as average. About 18% of the participants currently use any smartphone application to do health screenings. Among those who use these applications, 62.5% used the applications for free and stated the following problems when using them: interface issues, in-app purchases, accuracy, unnecessary advertisements, and network issues. In general, many of the participants (95%) were interested in using camera-phone applications for early detection of oral lesions to screen their family members about 3-4 times per year. Moreover, about 47.5% of participants thought it was of high importance to follow-up patients with a habit or history of detected oral lesions.

### Qualitative results from focus groups and in-depth interviews

The e-survey results informed how we framed the questions in the focus groups and in-depth interviews. Issues that required further elaboration were explored in a discussion format.

### FGD#1 findings

#### Topic A: oral health awareness

The focus group discussions suggest that the family members do not pay significant attention to oral health. This is exacerbated by a general lack of awareness about oral health, with young people themselves having minimal oral health information which was primarily obtained through dental visits. Many participants mentioned the resistance to discussion of oral health and oral cancer, especially among those who consume tobacco on a regular basis. There is a prevalent feeling of embarrassment around oral health which prevents people from accessing oral health providers until the situation worsens. The participants agreed that a mobile app which can deliver information about oral health and oral cancer will increase awareness and enable family members to access the information at their own pace.

#### Topic B: oral health behavior

There was no culture of regular dental checkups within the families in urban areas and a lack of adequate healthcare services in rural areas. The family members noted they would go to the dentist only if they were in pain and often avoided follow-up visits if the pain receded.

“[Family members] are ready to take the initial treatment but after that if there is relief, they would not want to go [back to the dentist]. In case their problem comes back again then they will go.”

In case of mouth sores, family members would often wait for a few days and try home remedies. If the sore did not heal, only then would they visit a dentist.

#### Topic C: advantages and disadvantages of designing an oral health mobile app for young people

Participants agreed that the involvement of a young person in operating mobile apps is beneficial. Participants from rural areas mentioned that discussing oral health with their family members is not an issue but involving them in using the mobile app was difficult due to lack of habit around technology use, lack of accessibility to smart phones and issues with internet connectivity issues. Participants from urban areas indicated it would be relatively easier to discuss oral health and to use a mobile app with their families. The participants mentioned the importance of having credible information on the app and suggested the app have “certification and authorization” to make their family members trust the mobile app. Participants mentioned that younger people were better equipped with skills to use new technologies. However, they also noted that convincing older adults for regular screenings may be difficult due to limited agency in the households.

“For us [young people] to be involved is a way better option because we know technology more as compared to our older generation. Usage of the app would be easier for us, and it will be easier for us to explain to them also how to use it. It is not important for like the app has to be used by the individual itself? You said the younger generation in the whole family can use the app and take pictures of the other people as well. So, it is just going to be voluntarily involvement of the family members. We are responsible for whatever we are clicking the pictures and all the activity that we are doing. It is just we have to convince them. Convincing them would become easier if we are able to explain them the benefits and also the usage, the results that we can get out of it.”—Young Person

#### Topic D: potential challenges with using an oral health mobile app

In our discussion we asked young people if they had heard about a new product that uses an app to help detect oral lesions, and if they could learn to use it to look for oral lesions in their family, would they be interested in trying it. All participants were open to the idea of using the mobile app but highlighted several issues that need to be considered. These included –

Fear around positive diagnosis: Some participants were curious to understand what would happen if a person received a positive diagnosis for a potentially malignant lesions after screening the oral cavity. They noted people might panic and discontinue using the app.
  “Also, they have the fear, what if they scan and it turns out to be positive and by not getting scanned it will be better… in ignorance. People are scared about what to do if they turn out to be positive. People tend to go in depression about how they will run their family, etc.” –Young Person
Possibility of deleting the app when the screening result is negative: Some participants highlighted people might delete the app when they receive a negative diagnosis after screening the oral cavity for malignant lesions thereby not solving the purpose of the app as a source of information on oral cancer prevention over a longer period.
Lack of accountability: Most participants raised the issue that app users would feel lack of accountability with app-based screening and instead would prefer to rely on a health care provider.

“If we are screening through an app, people might feel that there is no sort of accountability with the app and rather get screened in a professional setting.”—Young Person (FGD1)

#### Topic E: expectations from an oral health mobile app

Young people were asked what should be done to make such a mobile app interesting and effective for people to use for oral health awareness and early-stage oral lesion detection. The discussions highlighted several features which could be considered for the mobile app as documented in Table 1.

**Table 1:** Requested features for a mHealth application.

Topic	Features	Sample quotation
Ease of Use	Easy app design that enables use for both among older and younger people. Detailed instructions on how to click clear pictures of the oral cavity for screening Simple language making it easy for majority of the people to understand Positive framing of information around oral cancers to overcome fear and stigma Inclusion of stories, testimonials, quizzes, ratings, and animations Audio box in local language to help those users who are not comfortable typing Ability to switch between languages (English and Hindi to start) Periodic notifications to help people remember to check the apps and conduct screenings Gamification format to make the app interesting	*“We would require an interface which is accessible by a lot of people. We're thinking of getting a lot of samples and to make it more widely accessible, then it should be fairly simple, but should still be informative.”* *“I believe if it's built in a very simple manner rather than being complex, then it won't take a skill to do that.”* “*You need to create a positive environment around it… give them a new perspective that this can help you in the future … it's not necessary that you have cancer … We need to create a positive atmosphere.”*
Information access and security	Be able to use the app in offline mode to address issues of poor internet connectivity Ensure privacy and safety during screening process in the app Inclusion of a short diagnosis report in lay language Creation of a private chatbot for emotional safety and FAQs Users could feel they are part of a community (i.e., a place where people could ask their questions and get AI-generated answers). If additional information is required, then the option of a private chatbot could be presented.	*“When we use Google to get help for some disease related queries, it makes it overly complicated and that is why people hesitate in using technology because they feel things are made complicated and negative. So, there should be a private chatbot wherein privacy is maintained, and they can ask their own questions from AI and the AI can answer those accordingly.”* *“Selectively filtering out the information what people will want rather than just giving the entire information for oral cancer. You know, like, people can ask what are the steps that can be taken to improve our oral health or what is oral cancer to simplify information.”*
Collaboration with health care professionals	Allow for direct communication with health care providers Healthcare providers should recommend the app Provide information on hospitals/clinics where people could go for oral screenings Address fear around positive diagnosis by collaborating with HCP, including psychologists and counselors, to establish an emotional security net.	*“Maybe we could partner with hospitals and doctors, maybe they can inculcate trust, and you know take that method of detection in hospitals. That's how we can build upon credibility of the app."*

### IDI#1 with health care providers (Hcp) findings

We conducted a total of six in-depth interviews with community health workers (CHWs, *n* = 3) and physicians (*n* = 3, i.e., dentist, periodontist, and surgeon). The CHWs are volunteers with ICS, New Delhi. The physicians we interviewed are either practicing at ICS's Cancer Detection Center or in Maulana Azad Dental College which is a government institution in New Delhi.

#### Topic A: oral health knowledge

HCPs noted lack of awareness and knowledge related to oral health in general, and tobacco products (particularly bidis, hookahs, and e-cigarettes) and alcohol use as causes of oral cancers in the community.

“[People] replied positively about cigarettes [causing oral cancer] and said that it is not a good thing. When I asked them about other things besides cigarettes, so at that time those people were not aware of bidis [tobacco rolled in a tendu or temburni leaf]. They had the mindset that bidi is not harmful because no one raises any concern about them, so it is safe. We told them that it is not safe, and it is more harmful [than cigarettes]. It does not have filters like cigarette and secondly one needs to inhale [the bidi] deep for it to reach directly to the lungs. So, it is more harmful as it has more tobacco. Since they do not see any information about it, for them it is not harmful.”—Community Health Worker

HCPs noted several attractions to smoking and chewing tobacco. These include peer pressure, normalization of tobacco use among families, media influence, and stress factors.

“Initially, they [young people] find it very cool and secondly it is peer pressure. Peer pressure plays a very big role although everyone is aware that tobacco is not good for their health. They deliberately try it knowingly; they take it as a challenge specially it is there in men mostly because they have more tendency of getting and accepting challenge.”—Community Health Worker

“Because all the grandmothers and other elders are consuming it [chewing tobacco].”—Community Health Worker

We further asked HCPs how oral health is discussed within families. They mentioned how people largely focus around brushing teeth and rinsing the mouth after meals as a means of maintaining good oral hygiene. Besides lack of awareness, most HCPs mentioned accessibility and affordability as barriers to seeking oral health care.

“Oral health over here in India is the least of concern because first of its unaffordability… mostly it is costly. So, patients over here think at that level. I mean what more can happen if a decayed tooth comes later, you have to just get it extracted, but they do not know what a decayed tooth can cause. It cannot only cause tooth pain but it can cause an ulcer which can later go on to become cancerous and this is also related to even heart problems. I think the education level and for the normal people is really low.”—Physician

#### Topic B: oral health awareness and communication

HCPs were asked where people most get information about oral health. According to them, sources of credible information included doctors/dentists, awareness campaigns and television.

“On T.V. it is shown frequently that such things [tobacco products] should not be consumed. They show how cheek muscles are damaged after consuming pan masala [chewing tobacco], veins are damaged, blood is oozing out. Generally advanced stage is shown in that.”—Community Health Worker

Whereas sources of bad information or misinformation on oral health included family or social circle, informal health care providers, movies, and social media.

“[Source of bad information on oral health] that is within the family and by friends, they do not go to a doctor as such but what the friends say, they talk it out with their friends and what they say is what they take into consideration”—Physician

“The quacks working in small colonies, people visit them and they neither prescribe any diagnostic test nor provide any information. They only prescribe some medicines which provides temporary relief… this makes the patient believe that they are healed now.”—Community Health Worker

Many of the recommendations included reducing tobacco consumption and increasing awareness by educating, counselling, promoting discussions in community and interpersonal spaces about screening of oral cavity and early detection of cancerous lesions.

“People think that since they do not consume tobacco, they are not susceptible to oral cancer, but it can happen to anyone. We make people aware about how to identify signs of oral cancer even when you have no issues at all. Nobody looks into their own mouth. There are few people who look inside while brushing. We look inside our mouth when there is some pain, and we look for any swelling. But, without any problem, we do not open our mouth. We explain to people that twice in a week one should inspect their mouth while brushing.”—Community Health Worker

#### Topic C: likelihood of use of an oral health mobile app by young people

HCPs were asked what they thought about the idea of targeting young people by educating them on oral health awareness and encouraging them to conduct screening of the mouth cavity for their older family members. All HCPs were supportive of the idea. Although physicians thought the approach would work of engaging young people in conducting screenings, they questioned the long-term use of the app by young people and especially those belonging to socio-economically disadvantaged communities.

“There will be an impact because youngsters of today's time are very strong. At their age, we used to be suppressed by our elders, but this new generation has strong convictions. When youngsters go in groups, they easily convince people and people follow it also.”—Community Health Worker

“[It is] a very good approach, but then how much time will they [young people] give for it [the app]. This is a matter of concern because they have their own life. They are no doubt tech-savvy but how serious are they for this issue [oral health] is a major concern.”—Physician

#### Topic D: information to include in the mobile app about oral health

Nearly all HCPs mentioned that the mobile app needs to be interesting for it to be downloaded and used by people for oral cavity screening. The mobile app should cover information on risk factors and symptoms of oral cancer, impact of secondhand smoke, benefits of screening and early detection, address misinformation related to tobacco smoking and provide resources for tobacco cessation. One of the CHWs noted that besides including information on the harmful effects of smoking cigarettes and chewing tobacco, it would be helpful if the app included information on the use of hookahs and e-cigarettes as well.

“I have observed that the main focus in social media, print media, and mass media is that if a person smokes, the effect is on same individual. There is no mention of people around [the smoker] getting affected”—Community Health Worker

“There should be information on ways of prevention like early detection [and] screening. If that is there, people will know that it can be prevented and further it will not reach advance stage [fear of facial deformity]. They will know that they can heal by taking oral medicine and this sickness will not go further to advance stage.”—Community Health Worker

According to the HCPs specific features like educational videos and testimonials from celebrities who quit tobacco or oral cancer survivors could be added. Few health care providers mentioned that the mobile app should focus primarily on oral health (and not oral cancer) due to fear and stigma related to the word cancer. Moreover, the information on the mobile app around oral lesions should be framed in an optimistic tone to encourage people to go for oral cavity screenings.

“When we go for screening [or] when we say, it is an oral cancer screening [camp], there are many people who drop out. They say, we do not want to do [screening]. They are scared, the stigma is always there. But when we say this is a comprehensive health check-up, that is when the crowd comes.”—Physician

“[The mobile app should] start with an optimistic attitude the one can survive [oral cancer], one can avoid disfigurement if they go for treatment. Do put the word ‘if’ later on. ‘If’ go for the treatment at earlier stage and ‘if’ you delay, then this is the result. People might follow it and go for screening. People are scared of disfigurement more than death.”—Community Health Worker

#### Topic E: referrals and treatment

We asked HCPs where they would refer a patient for a thorough oral health assessment. Commonly, a dentist or a health clinic/hospital were mentioned. Most of the HCPs also mentioned cancer screening camps organized by ICS held every month in various parts of New Delhi. HCPs mentioned if a suspicious lesion is detected, the patient is referred to a hospital in their network for further assessment. In general, the barriers to seeking care mentioned by the HCPs included fear of diagnosis and taking time-off work as it results in the loss of day wages.

### FGD #2 findings

After completing a review of the wireframes, the student responses were tabulated by requested features and planned modifications. Requested features were discussed with the development and design teams and a collective decision was made to either implement the change immediately, to hold the suggestion until additional funds could be secured or to table the suggestion as too difficult to implement or inappropriate due to a misunderstanding as to the function of the app.

Students found the design easy to navigate and they appreciated the incorporation of additional privacy features to limit access to shared pictures and the medical history. They concurred that presenting the educational information in short, animated video format with multilingual support and closed captions was preferable. They also liked that the appointment reminders could be via in-app messaging, SMS text, or email, depending on the users' preferences.

We made the following changes to the final design: (1) included the option to not state the gender; (2) added some pictures of relevant lesions to the resources for educational purposes; (3) made additional assurances as to privacy in the onboarding section; (4) added a video tutorial on how to take the pictures; and (5) added in-app reminders for scheduled appointments. Some requests delegated to be considered at a later date included (1) employing a Bluetooth-enabled camera; (2) creation of multiple profiles under one account; and (3) exploring alternative solutions for image capture.

The students still expressed some reservations with the number of pictures (*n* = 13) required to fully scan the mouth and possible technical challenges related to the image taking but in consultation with our clinical advisor, we retained the 13-image design. Some students also aired some doubts as to whether their elder relatives would find the technology credible. This will need to be explored in more depth during the pilot testing of the application.

### IDI #2

HCPs found the app design to be user-friendly and thought it could help in detection on oral lesions. The HCPs preferred a demonstration of the OC-DETECT application with few patients before testing out the app in a larger sample. The HCPs thought including an appointment and message notification feature was useful for both patients and the HCPs to plan out in-person visits as well as communicate with the patients. The CHW highlighted that app navigation should be available in other regional languages as well for better uptake.

We implemented several suggestions in the final design that dentists highlighted as highly desirable such as (1) annotation capabilities; (2) the ability to zoom in on the images; and (3) the ability to log out of the account to maintain a high security level. Additional image editing and scaling features that could assist in making a final diagnosis for the patients were postponed until additional funding could be secured.

### Design round #2

Upon completion of the second round of FG and IDIs to gather feedback from key stakeholders, the IDP team made modifications to the Figma wireframes and delivered the design to the OC-DETECT research team. The international research team met repeatedly to review the revised design and made recommendations for additional modifications.

### Educational materials

After reviewing the e-surveys, in-depth interviews with the HCPs and initial focus group session transcripts, we identified five key topics for public education versions to be shown in the mHealth application (see Table 2). The micro-learning content was developed in both English and Hindi to embrace diverse cultural perspectives. To enhance user understanding, the language used in the content has been tailored to align with the Flesch-Kincaid (56, 57) readability level appropriate for 10 to 11-year-olds. This adaptation ensures that the educational materials are linguistically inclusive and comprehensible to a wide audience.

**Table 2:** Microlearning video topics embedded in the OC-DETECT mHealth application.

Video titles
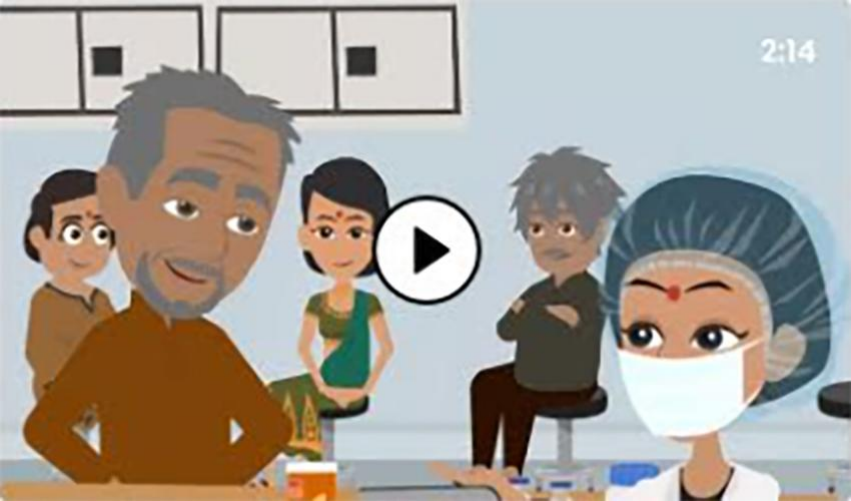
What to expect at a screening for oral lesions—Parts 1 & 2
Benefits of Early Oral Lesion Detection
Changes to the Mouth Linings Due to Smokeless Tobacco Use
Overview of Using the Checklists and Photos in OC-DETECT
How to Talk with Your Elder About Participating in a Research Study

### Final prototype design

**Figure 2:**
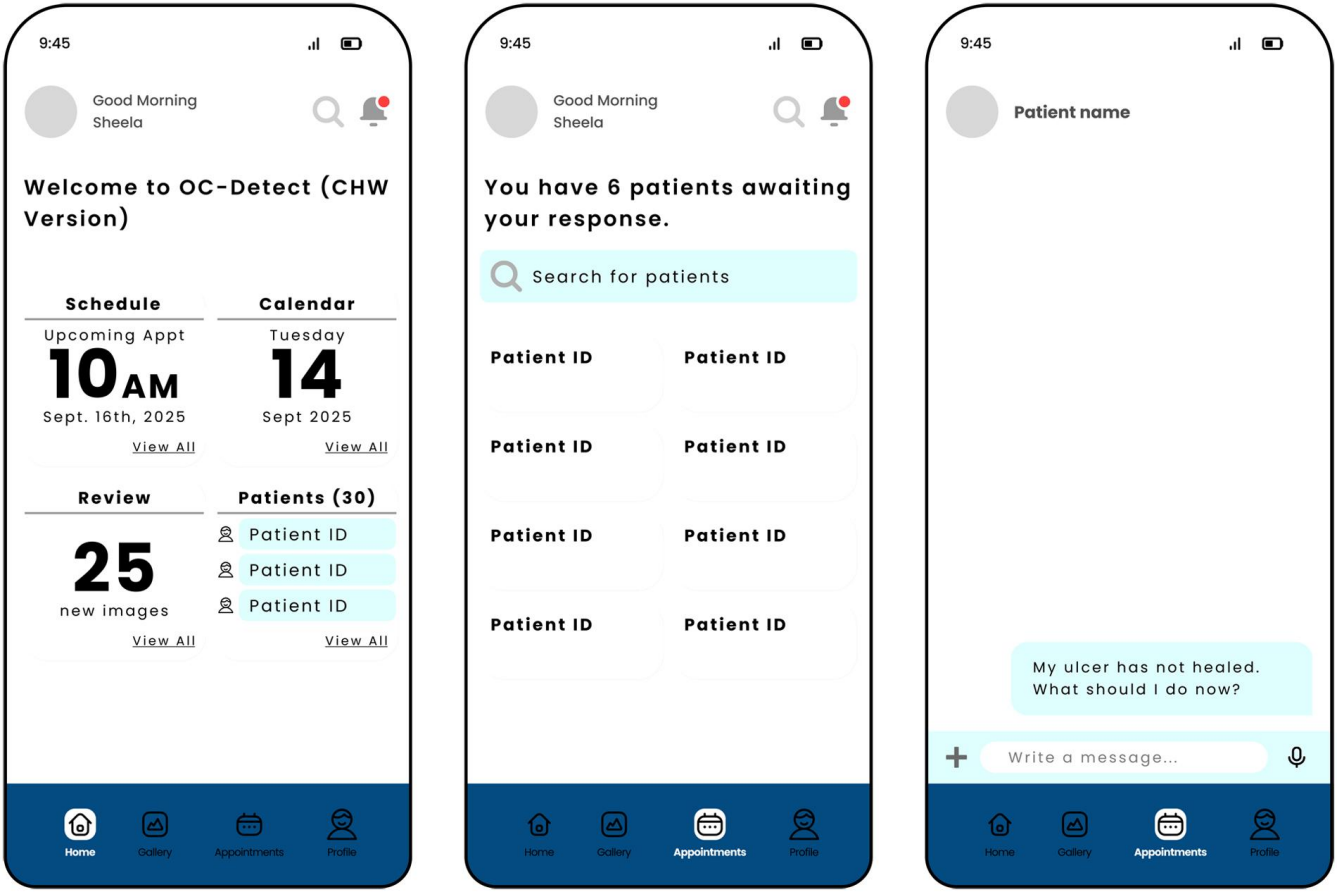
Representative wireframes presented to the young people during the focus groups.

After reviewing the e-survey, FG and IDI materials, the research team decided on the three designs for prototype testing, one each for the young person, clinician and nurse. For the young person, an mHealth application (app) was developed to collect demographics, complete health screening questionnaires, take 13 images of the oral cavity with a mobile phone, schedule appointments, review educational materials and interact with the nurse, if necessary (see Figure 2 Young Person Wireframes).

**Figure 3:**
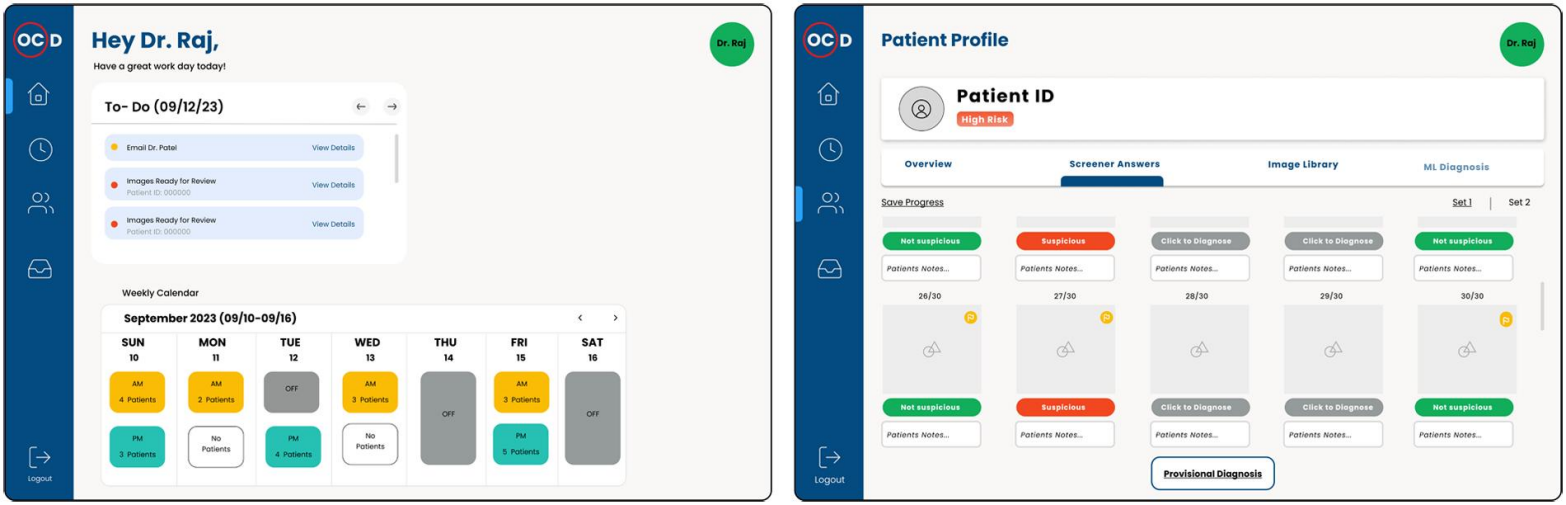
Representative wireframes presented to the clinicians during the interview.

For the clinician, a secure web-based application was designed to streamline patient review and follow-up coordination. The platform allows clinicians to view each participant's screener results, examine the uploaded oral-cavity images, and provide a provisional diagnosis for each image. Once reviewed, the system automatically notifies the participant through the app when an in-person appointment is advised (see Figure 3: Clinician Wireframes).

**Figure 4:**
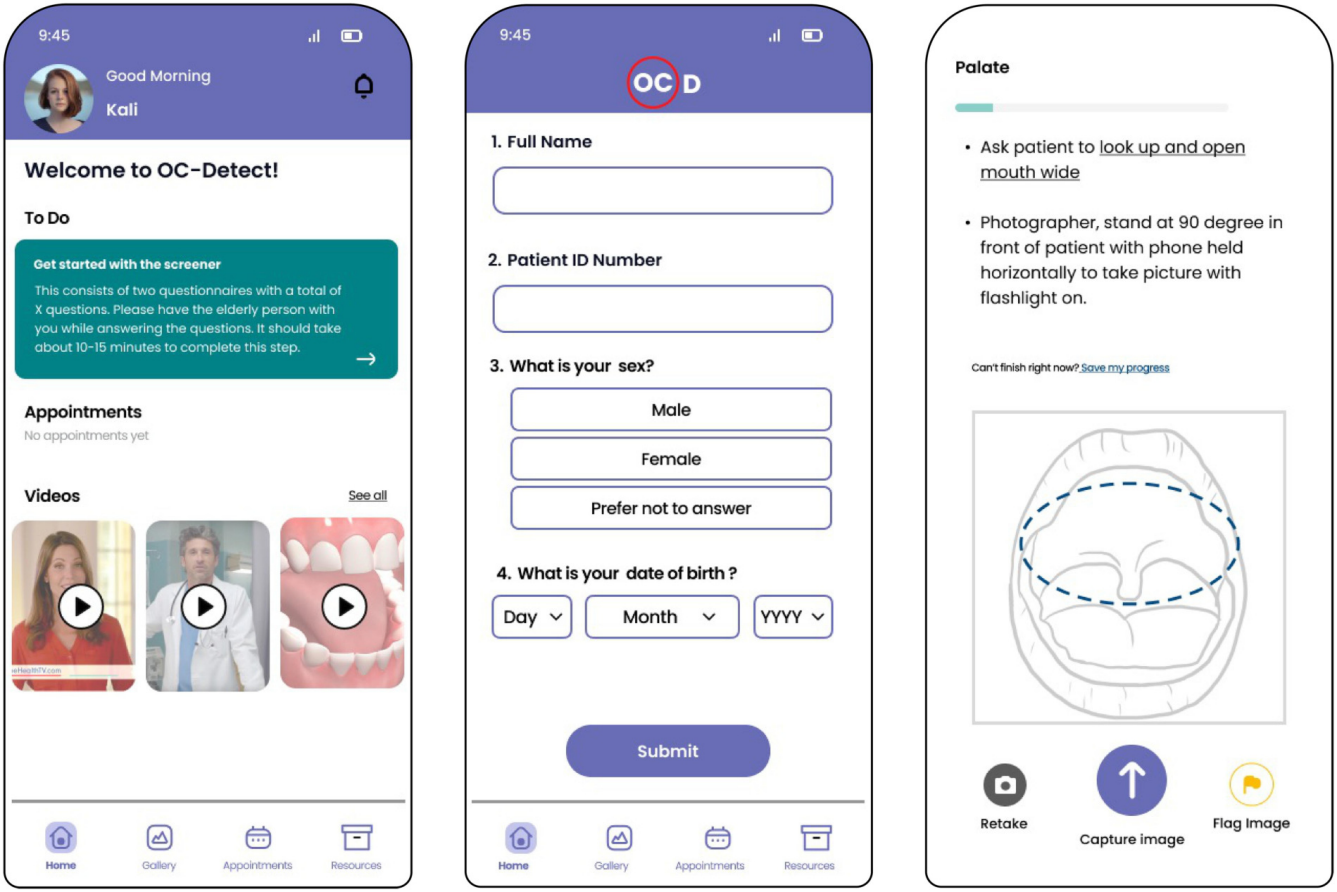
Representative wireframes presented to the nurse during the interview.

The nurse's mHealth app was created to review patient screening and image analysis results, access the educational content, coordinate appointments, and encourage follow-up via in-app messaging (see Figure 4: Nurse Wireframes).

The development team is employing an Agile methodology to iteratively refine the OC-DETECT platform through a series of focused sprints, each addressing a specific functional area. This approach enables progressive testing and feedback integration, ensuring that the prototype is technically stable and user-validated prior to pilot deployment.

In response to concerns expressed about security, we implemented privacy safeguards, including user authentication, encrypted data transmission, and secure cloud storage of de-identified images that were not saved to the user's personal photo gallery. Access to images was designed to be restricted to authorized study clinicians, and images were linked to study IDs rather than personal identifiers whenever possible.

## Discussion

Prevention, early detection through screenings, and timely and appropriate referrals to treatment are the most effective approaches to reducing OC morbidity and mortality (27, 35). According to a recent WHO 2025 report, over 380,000 new cases of oral cancer are identified each year globally, and there are over 180,000 deaths each year (58). Although India has one of the highest incidences of oral cancer in the world (59), the national screening rates there are low (60), and are especially low in the Delhi state (14.6% in urban and 6% in rural areas), for an overall regional average screening rate of 9% (10). The final prototype design of the OC-DETECT platform included features: (1) to assess participants for their risk for oral cancer based on their demographics and answers to a health care screen; (2) to collect images of the oral cavity using a mobile phone for remote analysis; and (3) to provide linkage to care at local clinics or dental camps. The creation of a mHealth application that can screen for health history, as well as tobacco and alcohol habits, combined with oral cavity imaging at home is hypothesized to address the problems of access as well as stigma and fear that likely contributed to these low screening rates.

E-surveys, focus group sessions, and in-depth interviews with potential end users and key stakeholders informed our prototype mHealth application entitled OC-DETECT. E-surveys and interviews with HCPs revealed that many HCPs believed lay people could master the at-home screenings and be instrumental in completing those screenings, especially with the aid of a younger family member who was tech savvy. In addition, HCPs indicated that many patients get their information from peers, family members and social media, which may be contributing to the lack of understanding of the value of early detection. The HCPs further suggested that a lack of awareness of the importance of maintaining oral health was a barrier to seeking medical care and the low screening rates.

The e-survey and focus group discussions indicated young people were aware of the dangers of SLT use and heavy alcohol use, and their link to oral cancers, and they knew that these cancers could be prevented, or after early detection, successfully treated. Considering this awareness, it was surprising to see that oral health awareness was still considered a problem in the community at large. As the young people surveyed in this grant were all enrolled in college, this may be an issue of education or socio-economic variables impacting the perceived general public's awareness. Future studies will need to survey a broader population.

As a direct result of the discussions in FG about stigma and fear, we opted to share the results of the risk-assessment in the app immediately (low or elevated risk) but to NOT inform the participants of the results of their image analysis reviews until they came in for an in-person exam with a physician. The reasoning was that this is a new technology and we did not want to release untested information. But we decided we will add a channel for the app users to reach out to a nurse for advice and we will plan to provide a Frequently Asked Questions (FAQs) section to allow users to do their own investigation through curated, evidence-based resources. We also plan to include (in the app) guidance on the number of screenings that should be done per year, as well as basic information on oral health care. We will create multiple animated videos (on topics on oral health, oral lesions, and what to expect at an oral health exam). These videos will be embedded in the app so no Internet connection will be required to view them.

Health education interventions can play a vital role in raising awareness about oral cancer and would encourage at-home oral examinations for early detection (36). By incorporating educational materials in both video and reading formats in both Hindi and English, targeted to the appropriate educational levels of the at-risk individuals, it is hoped that it will be possible to increase the number of screenings and provide opportunities for early interventions. The prototype will be pilot tested with 20 dyads, consisting of a younger person who is technologically savvy with a smartphone and an elder relative who is at-risk for oral cancer based on their smoking and alcohol habits. While the elder is waiting for the in-person appointment, the younger person can help the elder review the educational materials in the app.

Technology-assisted oral cancer screening and early detection have been implemented and validated in low-resource settings as a proof of concept. The primary aim of these prior approaches was to empower the community health workers. Studies have shown that trained community health workers can effectively screen for oral cancer using mobile phones, achieving 94.3% sensitivity and 99.3% specificity, comparable to physician diagnoses (21, 61). The current study confirms that family members are willing to take on the role of administering risk assessment screenings and image taking for their at-risk family members, and as such has the potential to alleviate the workload of the CHWs. Involving CHWs in both the distribution and the implementation of the app with the dyad is hypothesized to address concerns related to low digital health literacy, and the need for sustained engagement and integration with existing health systems.

Both students and healthcare providers expressed concerns about patient privacy. We incorporated into the design privacy safeguards, including user authentication, encrypted data transmission, and secure cloud storage of de-identified images that were not saved to the user's personal photo gallery. Access to images was restricted to authorized study clinicians, and images were linked to study IDs rather than personal identifiers whenever possible. As a direct result of the clinician's feedback, we created a logout feature for the provider's desktop view so no one could access the data if the provider stepped away from the setup. Future iterations could incorporate more formal privacy-by-design principles, including data minimization, clearer in-app consent and data-use transparency, on-device pre-screening to reduce uploads, and will reflect alignment with emerging digital health and data-protection regulations in the target regions. In addition to improving the underlying security, we anticipate bolstering trust and acceptance of the app by using CHWs to serve as the liaison between the healthcare providers and the family members.

The low reported patient compliance for biopsies in other studies highlights the need for a minimally invasive diagnostic methodology. Alternative diagnostic methods, involving AI models combined with white light or auto-fluorescence or biomarkers have yielded some promising results. White light - AI models detected precancerous lesions from photos with an accuracy score of 0.84 (62). Adjunct diagnostic tools like AI-enabled, dual-mode imaging (white light and auto-fluorescence) have shown 87% sensitivity and 86% specificity (63). Biomarker-based oral cytology, a minimally-invasive method, has proven to be alternative for invasive biopsies. A recent study found that biomarkers (Cyclin D1, CD44, MAA, SNA-1) can accurately detect high-risk oral lesions with 89% sensitivity and 92% specificity in identifying high-grade dysplasia and oral cancer (64). Currently, however, none of the tools available for early detection of oral cancer have been implemented for use by the general public, suggesting the need to move the technology into the hands of younger people already equipped with smartphones and the desire to ensure their elders' health. Further research will need to focus on whether or not this technology can be used in populations with low digital and traditional literacy skills and test options to adapt this technology in these populations.

## Limitations

This study was restricted to participants in urban settings, specifically New Delhi, due to time and resource limitations, and convenience sampling was used. As such, we may have introduced a potential selection bias. In addition, as with any focus group session with multiple attendees, a social desirability bias may have been introduced, affecting the accuracy of the findings. Many of the younger individuals who participated in the e-surveys and focus groups attended a university in New Delhi and represented a more educated group than typical of the general population. As such our study population was not representative of the end user population, individuals in rural communities who may have older phone systems and low Internet access. But as image capturing of oral cavities is a relatively new technology, we wanted to focus at this stage on creating an easy-to-use interface. In subsequent research we will work on ways to adapt the technology to low Internet resource settings and low digital literacy populations. Other limitations are that the initial health screenings are self-reported and therefore subject to bias.

## Conclusion

The potential for mHealth screenings to save lives and reduce health care costs is vast considering the prevalence of SLT and tobacco use (26%) (65) and the population of India (1.4 billion in 2025). With the telecommunications infrastructure in India transitioning to 5G, there is potential for the product in India as well as other low and middle-income countries (LMICs) to improve population surveillance and screening programs and thereby lower mortality due to OC. Future research can focus on using teledentistry services to avoid time lost to travel back and forth to clinics and to provide additional privacy by providing an initial consultation from the home. Once commercialized, OC-DETECT will have the ability to empower and educate at-risk populations and, in so doing, improve rates of prevention screenings and early detection of oral cancer, thereby reducing morbidity and mortality rates in India, LMIC countries and rural regions of the United States. Employing formative research with a user-centered focus early in the design process can reduce production costs and enhance feasibility and acceptability of the final prototype design.

## Data Availability

The raw de-identified data supporting the conclusions of this article will be made available by the authors, without undue reservation.
