# Supplementary material for: Formative research and design for a mobile health platform for oral cancer screening and detection (OC-DETECT)—a mixed methods study
Source: Front Digit Health. 2026 Feb 2;7:1738874. doi: 10.3389/fdgth.2025.1738874 (PMC12908169; doi:10.3389/fdgth.2025.1738874)
Supplement: Supplementary file 1 [file Datasheet1.pdf]

SUPPLEMENTARY TABLES

Supplemental Table 1: Indian Healthcare Provider Responses from an E-Survey (N=11 respondents)

**Do you think a lack of oral health awareness is a problem in your community?**	**Number**	**%**
Yes	11	100%
No	0	0
Total	11	100%
**How many times a year would you screen a patient over 40 years of age for oral lesions?**	**Number**	**%**
Annually	8	72.73
Biannually	1	9.09
Quarterly	2	18.18
Total	11	100
**What are perceived reasons for oral health being under-addressed?**	**Number**	**%**
Lack of oral health awareness	5	45.45
Lack of concern for oral health	4	36.36
Others (Necessary for all/we see many patients)	2	18.18
Total	11	100
**Do you think that young family members can use mobile app to screen for oral lesions?**	**Number**	**%**
Yes	10	90.91
No	1	9.09
Total	11	100
**Why or Why not?**	**Number**	**%**
Technology has made many things easier	3	33.33
[Young people] are more tech -savvy	3	33.33
At home can't provide full facilities	1	11.11
Since young generation is hooked on mobile and apps, providing an app along with its usage will help them do self-screening	1	11.11
Hygiene	1	11.11
Total	9	100

**What is the biggest barrier in seeking help for oral lesions?**	**Number**	**%**
Lack of awareness	5	45.45
Lack of facilities and no awareness	3	27.27
Others (no priority/tobacco/etc.)	3	27.27
Total	11	100
**Do you think there is need of awareness around oral health, cancer prevention and treatment?**	**Number**	**%**
Yes	11	100
No	0	0
Total	11	100
**Do you provide patients with materials on oral health?**	**Number**	**%**
Yes	7	70
No	3	30
Total	10	100
**Where do people get wrong health information from?** (Open-ended responses)	**Number**	**%**
Peer, friends, relatives, social media	6	60
Advertisements	1	10
No [people do not get incorrect health information]	1	10
Informal health care providers (e.g., quack doctors)	1	10
Villages	1	10
Total	10	100
**What are the reasons for past tobacco campaigns being unsuccessful?** (Open-ended responses)	**Number**	**%**
Lack of awareness and information	4	40
Addiction	2	20
It is totally discouraging	1	10
Lack of political will. Lack of Tobacco Control measures. Ignorance of people because they see parents and relatives using tobacco. Sale of single sticks of cigarettes. Cheap pouches of chewing tobacco.	1	10
The lucrative advertising by tobacco companies overshadowing the campaigns	1	10
Successful [Past campaigns were NOT unsuccessful]	1	10

Supplemental Table 2: Young Persons' Existing Oral Health Knowledge (N=56 respondents; Note, multiple responses were possible, so, the total may be more than 100%).

**What would you say are the causes of lesions in the mouth?**	**Number**	**%**
Tobacco use (e.g., cigarettes, chewing tobacco, gutka, areca nut, etc.)	39	70%
Heavy alcohol use	15	27%
Sexually transmitted virus called human papillomavirus (HPV)	12	21%
Weakened immune system	9	16%
Excessive sun exposure to lips	3	5%
Other reasons	3	5%
Total	**56**	
**Do you think oral lesions can be prevented?**	**Number**	**%**
Yes	41	97.62
No	1	2.38
Total	42	
**Do you think oral lesions can be treated?**	**Number**	**%**
No	1	2.38
Yes	41	97.62
Total	42	
**Do you think lack of oral health awareness is a problem in your community?**	**Number**	**%**
Yes	39	92.86
No	3	7.14
Total	42	
**Where would you go to get credible information about oral health?**	**Number**	**%**
Doctors or Physicians	22	39%
Dentist	22	39%
Government hospitals	15	27%
Mass media (e.g., Newspaper, TV, radio, etc.)	15	27%
Social media	14	25%
Private Hospitals or Health clinics	12	21%

Professional organizations (e.g., World Health Organization, Indian Council of Medical Research, etc.)	7	13%
Non-government organizations	6	11%
Informal health care providers (e.g., Pharmacist, etc.)	6	11%
Ministry of Health	5	9%
Frontline health workers(e.g., Auxiliary Nurse Midwifes (ANMs), Accredited Social Health Activist (ASHAs), etc.)	4	7%
Nurses	2	4%
Other places	1	2%
Total	56	

Supplemental Table 3: Student Responses on an E-Survey on Mobile Phone Access and Connectivity in India (n=47 respondents)

**A. Hardware and connectivity**		
**Do you own a smart phone?**	**Number**	**%**
Yes	46	97.87
No	1	2.13
Total	47	
**Do you have unlimited data plan on your phone or tab?**	**Number**	**%**
Yes	32	68.09
No	15	31.91
Total	47	
**Do you have unlimited text messaging facility on your phone or tab?**	**Number**	**%**
Yes	28	59.57
No	19	40.43
Total	47	
**For your service plan on your phone or tablet now, do you pay as you go?**	**Number**	**%**
Yes	39	82.98
No	8	17.02
Total	47	
**Do you have access to wi-fi at university or home?**	**Number**	**%**
Yes	45	95.74
No	2	4.26
Total	47	
**Self-rating of the stability and reliability of the cell phone network reception (1=poor, 5=excellent)**	**Number**	**%**
1	0	0
2	5	11.11
3	16	35.56
4	14	31.11
5	10	22.22

Total	45	
**Self-rating of the coverage of the cell phone network connection (1=poor, 5=excellent)**	**Number**	**%**
1	0	0
2	3	6.82
3	17	38.64
4	14	31.82
5	10	22.73
Total	44	

Supplemental Table 4: Current Use of Any Health-Related Apps on a Mobile Phone by Young Persons in India (n=46 respondents)

**Do you currently use any smartphone applications to do any health screening?**	**Number**	**%**
No	37	82.22
Yes	8	17.78
Total	45	
Of those who responded 'yes' in the above question (n=8):		
**Are those applications free?**	**Number**	**%**
Yes	5	62.5
No	3	37.5
Total	8	
**What are the common problems faced while using those mHealth applications?**	**Number**	**%**
Interface, premium options	1	16.67
They are costly	1	16.67
Unnecessary advertisement	1	16.67
Accuracy	1	16.67
Advertisements	1	16.67
Sometimes a network issue	1	16.67
Total	6	

**If there was a camera-phone app that could be used in early detection of oral lesions, would you be willing to screen your family members for these lesions 3-4 times/year?**	**Number**	**%**
Yes	38	95
No	2	5
Total	40	
**How important do you think follow-ups are for patients with habit or history/ lesions detected? (1=low importance, 5= high importance?)**	**Number**	**%**
1	0	0
2	0	0
3	11	27.5
4	10	25.0

5	19	47.5
Total	40	
